# Process Development for Flexible Films of Industrial Cellulose Pulp Using Superbase Ionic Liquids

**DOI:** 10.3390/polym13111767

**Published:** 2021-05-28

**Authors:** Diana C. M. Ribeiro, Rafael C. Rebelo, Francesco De Bon, Jorge F. J. Coelho, Arménio C. Serra

**Affiliations:** Centre for Mechanical Engineering, Materials and Processes (CEMMPRE), Department of Chemical Engineering, University of Coimbra, Rua Sílvio Lima-Polo II, 3030-790 Coimbra, Portugal; ribeiromdiana@gmail.com (D.C.M.R.); rafael.rebelo.94@gmail.com (R.C.R.); francesco.debon91@gmail.com (F.D.B.); jcoelho3@gmail.com (J.F.J.C.)

**Keywords:** industrial pulp, cellulose films, superbase ionic liquids, IL quantification, mechanical properties

## Abstract

Due to environmental concerns, more attention has been given to the development of bio-based materials for substitution of fossil-based ones. Moreover, paper use is essential in daily routine and several applications of industrial pulp can be developed. In this study, transparent films were produced by industrial cellulose pulp solubilization in tetramethylguanidine based ionic liquids followed by its regeneration. Films were characterized by Fourier transform infrared spectroscopy (FTIR), scanning electron microscopy (SEM), UV/Vis spectroscopy, proton nuclear magnetic resonance (^1^H-NMR), dynamic scanning calorimetry (DSC), thermal analysis (TG), and X-ray diffraction (XRD). Mechanical tests showed that films have a good elongation property, up to 50%, depending on ionic liquid incorporation. The influence of the conjugated acid and dissolution temperature on mechanical properties were evaluated. These results revealed the potential of this methodology for the preparation of new biobased films.

## 1. Introduction

Most manufactured plastic products depend on oil-based feedstocks, with a steady rising trend due to their excellent mechanical, thermal, and barrier properties, as well as their established manufacturing industrial processes [1]. Despiteof the recycling efforts, such materials have shown their persistence in the environment as pollutants, while contributing negatively to the CO_2_ footprint, as their entire manufacturing process is extensively based on fossil resources. The alternative to this *status quo* is the introduction of biobased and biodegradable polymers to create a circular economy of greener materials supported by industrial processes.

Due to the enormous amount and variety of “petro-plastics” that should be replaced, cellulose, the most abundant biopolymer on Earth, may fulfill this task [2]. Cellulose is a linear homopolymer composed of D-anhydro glucopyranose units linked together by β-(1–4)-glycosidic bonds [3]. The presence of hydroxyl groups on the structure and the strong intra and intermolecular hydrogen bond network severely hamper cellulose dissolution in water and in common organic solvents [4]. This reduces the potential available applications without an *a priori* severe structural modification of cellulose. At the present time, only some “exotic” solvents or harsh chemicals can dissolve cellulose, like carbon disulfide (CS_2_), that is used in viscose process used at industrial scale [5], DMAc/LiCl [6], NaOH/Urea [7] and *N*-methylmorpholine-*N*-oxide (NMMO) [8]. Though, these solvents have severe drawbacks like intrinsic instability, toxicity, high flammability, as well as being difficult to recover or recycle.

Ionic liquids (ILs) have emerged in the last 20 years as a class of potential solvents for cellulose [9,10,11]. ILs are salts with a melting temperature below 100 °C, often liquids at room temperature. These solvents have a wide range of advantages like high (electro)chemical and thermal stability, near zero volatility (with negligible contribute to VOCs), high solvation capabilities and a tunable structure that can be modified according to the solubility requirements [12]. Earlier studies demonstrate that ILs are very effective to dissolve cellulose in high concentration (20–24 wt%) within short times (1–12 h) and without any cellulose pretreatment [13,14]. These solvents open a myriad of new options in the development of cellulose functional materials like composites, fibers, regenerated cellulose films [11,15] and hydrogels with enhanced mechanical properties [16]. Nevertheless, given that some ILs exhibit toxicity and some of them are difficult to recycle/recover, recently a new generation of ionic liquids has emerged. These ILs are obtained by combining an organic superbase like 1,1,3,3-tetramethylguanidine (TMG), 1,5-diazabicyclo-[4.3.0]non-5-ene (DBN), 1,8-diazabicyclo [5.4.0]undec-7-ene (DBU) or 7-Methyl-1,5,7-triazabicyclo[4.4.0]dec-5-ene (mTBD) with short chain carboxylic acids like formic, acetic or propionic acid [17,18,19]. TMG based ILs could be recycled via distillation, regenerating the superbase with recovery of 99% purity [18]. The ionic liquids dissolve cellulose due to the ability of the basic anions to break up the intra and intermolecular hydrogen bonds of cellulose by interacting with the hydroxyl groups of the glucose units [20,21]. The dissolution rate is unfortunately slow due to the high viscosity of the cellulose/ionic liquid solution, but it can be improved by the addition of co-solvents, like dimethyl sulfoxide (DMSO), *N*,*N*-dimethylacetamide (DMA), or 1,3-dimethyl-2-imidazolidinone (DMI) [4,22]. Co-solvency offers more flexibility because the physicochemical properties like heat, mass transfer and viscosity of the binary solvent mixtures can be varied continuously by changing the proportion of both components (ionic liquid and co-solvent) [10]. Distillable ionic liquids based on TMG have already been used in previous work to dissolve cellulose, but the possibility of film production has not been explored [18,23].

In this work, a new method for obtaining transparent films from the processing of an industrial pulp was developed. The method is based on TMG as a superbase conjugated with acetic or propionic acids. This solvent is more affordable than others base like DBN as superbase due to the lower cost of TMG. In this study the effects of the solubilization temperatures and the retained ionic liquid as plasticizer, on the film properties, were analyzed. This opens a new generation of cellulose-based materials made from a low cost and potentially recyclable solvent.

## 2. Materials and Methods

### 2.1. Materials

A sample of industrial cellulose pulp (bleached eucalyptus kraft pulp, BEKP, DP ≈ 1100) was kindly supplied by Celtejo S.A. cellulose industry. Microcrystalline cellulose (Avicel^®^, PH-101, ~50 µm particle size, DP ≈ 240, Merck KGaA, Darmstadt, Germany), 1,1,3,3-Tetramethylguanidine (TMG, 99%, TCI, Zwijndrecht, Belgium), acetic acid (TCI, >99.5%, Zwijndrecht, Belgium), propionic acid (TCI, >99%, Zwijndrecht, Belgium), formic acid (TCI, >98%, Zwijndrecht, Belgium), dimethyl sulfoxide (DMSO, 99.9%, Fisher-Sci,, Loughborough, UK), dimethylformamide (DMF, HPLC grade ≥99.5%, Fisher Sci,, Loughborough, UK), and deuterium oxide (D_2_O, 99.9% deuterium, Eurisotop, Cambridge, UK) were used as received. Deionized water was obtained by reverse osmosis.

### 2.2. Characterizations

Films were characterized by FT-IR in ATR mode using an Agilent Technologies Carey 630 spectrometer (spectrometer, Agilent Technologies, Waldbronn, Germany) equipped with a Golden Gate Single Reflection Diamond ATR in 4000–750 cm^−1^ range at room temperature. Spectra were collected with 4 cm^−1^ spectral resolution and 64 accumulations. ACD/Curve Manager software was used to process spectra. ^1^H-NMR spectra were recorded on a Bruker Avance III 400 MHz spectrometer (spectrometer, Bruker Corporation, Praha, Cz. Republic), with a 5 mm TXI triple resonance detection probe used to record ^1^H-nuclear magnetic resonance (NMR) spectra of cellulose solvent and extraction solutions of films, in D_2_O. The amount of TMG-based ionic liquid extract was calculated by integration of cellulose solvent pattern peaks using MestRenova software (v. 14.1.1, Mestrelab, Spain) (DMF intern standard). Thermal stability of the samples was studied by thermogravimetric analysis (TGA) that was carried out using NETZSCH STA 44F5 (Netzsch, Selb, Germany). Samples weights ranging from 5 to 10 mg were used in a temperature range of 20 °C to 500 °C at a heating rate of 10 °C·min^−1^, under nitrogen purge flow. Thermal behavior was evaluated by differential scanning calorimetry (DSC) performed in a NETZSCH DSC 204 F1 Phoenix model (Netzsch, Selb, Germany). All scans were analyzed in aluminum pans with an ordinary closed aluminum lid, in a dry nitrogen environment with a purge flow of 60 mL·min^−1^ and a heating/cooling rate of 10 °C·min^−1^. The samples were cooled from room temperature to −90 °C, followed by a heating cycle to 400 °C. The solvent volatilization temperature (Ts) and cellulose stability temperature (Tc) were taken as the peaks of the endothermic and exothermic phase transitions, respectively. All the values were collected from heat flux curve. Tensile tests were performed on an Instron 5944 (mechanical tester, Instron, Buckinghamshire, UK) equipped with a 250 N load cell. Cellulose film rectangular-shaped samples (50 mm; 10 mm; 0.1–0.15 mm) were submitted to tension at a rate of 5 mm·min^−1^ until failure. The Young’s modulus was calculated from the initial slope of the curve after removing the toe region. The values presented are an average of five valid tests.

To investigate the homogeneity of the TMG-based ionic liquid films, they were analyzed by SEM. The samples were frozen in liquid nitrogen prior to fracture to prevent the risk of plastic deformation. The surfaces were coated with gold and analyzed in a field emission scanning electron microscope (FESEM, ZEISS MERLIN, Oberkochen, Germany), ZEISS MERLIN Compact/VPCompact, Gemini II. To evaluate the transparency of films UV-Vis studies of the films were performed with a Jasco V-530 spectrophotometer (spectrophotometer, Jasco Europe, Cremella, Italy). The analyses were carried out in the 200–800 nm range.

To evaluate the crystallinity of cellulose pulp and regenerated cellulose films, X-ray diffraction (XRD) was performed using a powder X-ray diffraction instrument (X’PERT MPD, Philips, Malvern, UK). The XRD patterns with Cu Kα (0.179 nm) radiation at 40 kV and 30 mA were collected in the 2θ range from 5° to 50° at a scanning rate of 1.5° min^−1^. The crystallinity index (CrI, %) was estimated using Segal’s method [24,25] as follows:

CrI = (I_002_ − I_am_)/I_002_ ∗ 100, (1)

where I_002_ is the height of scattered intensity at the main peak, and I_am_ is the height of the minimum peak intensity.

### 2.3. Dissolution of Industrial Pulp

Raw industrial pulp (BEKP) was ground with a coffee grinder to get a fluffy material and maintained at *T* = 50 °C. The ionic liquid was obtained by acid–base neutralization and prepared by adapting a previously reported procedure [18]. Briefly, TMG (14 mL, 0.11 mol) was added to a round bottom flask and heated at *T* = 90 °C. Then, the desired carboxylic acid was added slowly and under vigorous stirring to TMG, to achieve a 1:1 molar ratio mixture. To reduce the viscosity, 10 mL of DMSO were added to the mixture as co-solvent. Thereafter, cellulose ground pulp (≈610 mg; DP ≈ 1100) or microcrystalline cellulose (Avicel^®^) (≈610 mg, DP ≈ 240) was slowly added to the solvent mixture under vigorously stirring to obtain a 2 wt.% (for BEKP) or 5 wt.% (for Avicel^®^) yellowish cellulose solution, which was left for 2 h at the desired temperature (90, 110, or 130 °C).

### 2.4. Preparation of Cellulose/TMG-Based Ionic Liquid Composite Film

**Figure 1:**
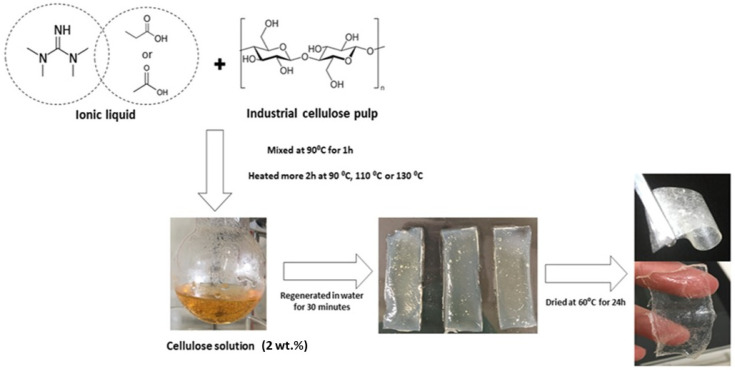
Schematic presentation of industrial pulp dissolution process and its film formation.

After dissolution, the procedure to obtain cellulose films consists of three consecutive steps: (1) heating cellulose solution (90, 110 or 130 °C), (2) water washing and regeneration, and (3) drying. First, to study the effect of temperature on film formation, cellulose solutions were heated at 90, 110, or 130 °C for 2 h. Then, cellulose solutions were cooled to 60 °C. To obtain the gel, solutions were spread on a glass plate into a six-slot silicone mold (2.5 × 6 × 0.3) cm and 300 mL of distilled water was poured in (cellulose/TMG-based IL solution: water = 1: 12.5 (*w*/*v*)), three times for 10 min each washing. A control experiment was done with a dissolved cellulose sample but using a large excess of water for regeneration (cellulose/TMG-based IL solution: water = 1: 50 (*w*/*v*)) during 24 h. This control results in the formation of a whitish paste due to the remotion of the whole IL and no film was formed. The mold was removed and gels were carefully dried with absorbent paper. Finally, to obtain films, gels were dried in a conventional oven at T = 60 °C for 24 h. During drying, gels were pressed (~100 kPa) between two glass plates. Figure 1 presents a schematic representation of film formation.

### 2.5. Quantification of Ionic Liquid in Films

The amount of ionic liquid retained in films was calculated by extracting formed dried films with deuterium oxide, for 24 h, with stirring (150 rpm) at room temperature, and the resulting solution was analyzed by ^1^H-NMR, using an internal standard (DMF). After extraction test, a FTIR spectrum was acquired to prove that all ionic liquid was extracted from film.

## 3. Results and Discussion

### 3.1. Cellulose Dissolution

Two types of cellulose were tested, industrial pulp (BEKP) and microcrystalline cellulose (Avicel^®^ PH-101). For cellulose dissolution, three different TMG based ionic liquids were tried. (1) TMG-acetic acid ([TMG][OAc]), (2) TMG-propionic acid ([TMG][Pr]), and (3) TMG-formic acid ([TMG][Fo]) (Table 1). [TMG][OAc] and [TMG][Pr] were the most effective in dissolving cellulose, contrary, [TMG][Fo] was not effective in dissolution because formate anion is a weaker hydrogen bond acceptor due to itsqw high acidity compared to the other carboxylates assessed through proton affinities [26]. *T* = 90 °C is the minimum for the development of cellulose solutions with adequate flowability. The solutions (TMGH^+^ + carboxylate anion + cellulose) exhibited a very high viscosity and to reduce it, DMSO, a polar aprotic co-solvent was added. This co-solvent accelerates mass transfer and diffusion and solvates the ionic liquid, forming more free ions and promoting the interaction with hydrogen bonds of cellulose [27]. The results are presented in Table 1. As expected BEKP, with a higher DP, is less soluble than Avicel^®^ [28] and [TMG][Fo] was not able to dissolve any of the cellulose samples.

### 3.2. Cellulose Films Based on TMG Ionic Liquids

There are few reports of using TMG ionic liquids for cellulose dissolution and none report the regeneration of the material to films formation. Besides that, the use of industrial pulp is rarely reported even though this material presents the most interest from an industrial point of view [15,29,30]. After cellulose dissolution at 90 °C the regeneration process with water was evaluated [31]. Film consistency largely depends on the process of washing/regeneration, being the amount of water and washing time the main variables of the process; the water used to regenerate was at ambient temperature, 18–21 °C. To determine the optimal washing conditions, several washing times were tested. The same amount of dissolved cellulose was poured in 6 silicon molds and 300 mL of water was used in the regeneration bath. This amount covered the mold with cellulose solution. It was found that 30 min (3 washing, 3 times 10 min each) resulted in clear and consistent gels. This combination of amount of water and time of each wash was used for the rest of work. Moreover, a shorter washing time, 10 min (two times 5 min), resulted in sticky, yellowish gels of low consistency. This could be due to the presence of a large amount of IL and their high plasticizing effect [11,32] and, to a partial cellulose regeneration. Contrariwise, long washing times of 24 h with several water changings, originates a brittle and whitish material, probably due to the absence of ionic liquid. This fact supports the idea that these kinds of films can be considered as a composite of cellulose and ionic liquid [33]. This optimization was performed by empirical knowledge, based on observation and several experiences made.

After the gel formation, a pressure-drying process (100 kPa, 60 °C) for 24 h leads to cellulose transparent films that were analyzed by FTIR, SEM, mechanical properties and TGA and DSC.

As cellulose dissolution implies the effective destruction of intermolecular and intramolecular hydrogen bonds and the effect of dissolution temperature is more discussed in relation to the kinetics of the dissolution process [34]. In this study the time of dissolution was constant but three different temperatures, 90 °C, 110 °C, and 130 °C, were evaluated. The capacity of the two types of cellulose (BEKP and Avicel^®^) to form films was analyzed. Films made with Avicel^®^ are always brittle and exhibited lack of consistency; BEKP, on the other hand, due to the higher DP, is more effective for film production, probably because of its bigger chains that can act as better polymeric support, and promotes the better retention of the ionic liquid [28]. As a result, only films made of BEKP were further evaluated and characterized.

The films were labeled as the acid used with TMG and the temperature of cellulose solution, i.e., a film labeled “Ac90” was a film that results from cellulose dissolution in a TMG and acetic acid ionic liquid ([TMG][OAc]) with a dissolution temperature of 90 °C. In all experiments DMSO was used as co-solvent to reduce viscosity and allow an easy film deposition.

### 3.3. Fourier-Tranform Infrared Spectroscopy

**Figure 2:**
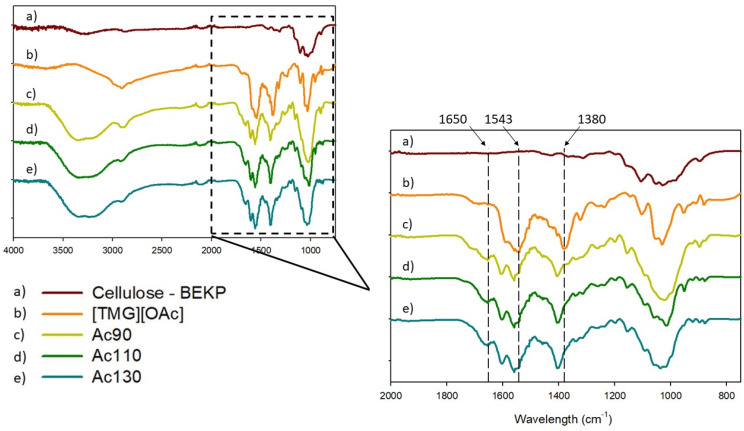
FT-IR spectrum of (**a**) BEKP, (**b**) [TMG][OAc], and films (**c**) Ac90, (**d**) Ac110, (**e**) Ac130.

FTIR analysis was performed to establish the chemical composition of films and understand the chemical changes in cellulose due to different solubilizing temperatures. The spectra are presented in Figure 2.

According to the literature, the hydroxyl group stretching vibrations at 3300 cm^−1^ and at 1650 cm^−1^ are due to the water absorption by cellulose, that are characteristic of the regenerated samples (Figure 2) [35,36]. The films show the presence of ionic liquid independently of the cellulose dissolutions’ temperature (90, 110, or 130 °C) (signals at 1543 cm^−1^ and 1380 cm^−1^). The shift of the methyl groups of the ionic liquid signal from 1380 to 1400 cm^−1^ is probably due to interaction with cellulose. An increase of temperature of dissolution does not cause any changes in cellulose structure and no new peaks appeared. This fact agreed with the literature that reports that TMG is non-destructing and a non-derivatizing solvent [18]. The FTIR spectra of films obtained from [TMG][Pr] are presented in Figure S1.1.

### 3.4. Scanning Electronic Microscopy

**Figure 3:**
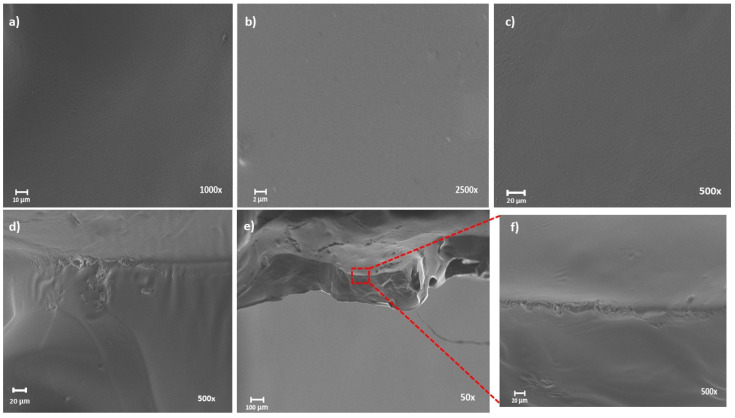
SEM images of cellulose films: (**a**) top view, Ac110, ×1.0 K; (**b**) Pr130, ×2.5 K; (**c**) Ac130, ×500; cross sections view, (**d**) Pr110, ×500; (**e**) Ac90, ×50; (**f**) Ac90, ×500.

The morphology of films was analyzed by SEM pictures of the surfaces (Figure 3a–c) and cross section fractures (Figure 3d–f). The surface of all films appears very homogeneous with no breaks or holes and no sign of fibrous structure is evident. This fact proves the complete regeneration of cellulose into another supramolecular arrangement, similar to other reported work [35]. Besides, the cross-sections of the materials show homogeneous and very compact morphology (Figure 3f) and two small holes on bottom (Figure 3e), that could be due to the fracture process, well. All films seem to be very similar in terms of morphology.

### 3.5. UV/VIS Spectroscopy

**Figure 4:**
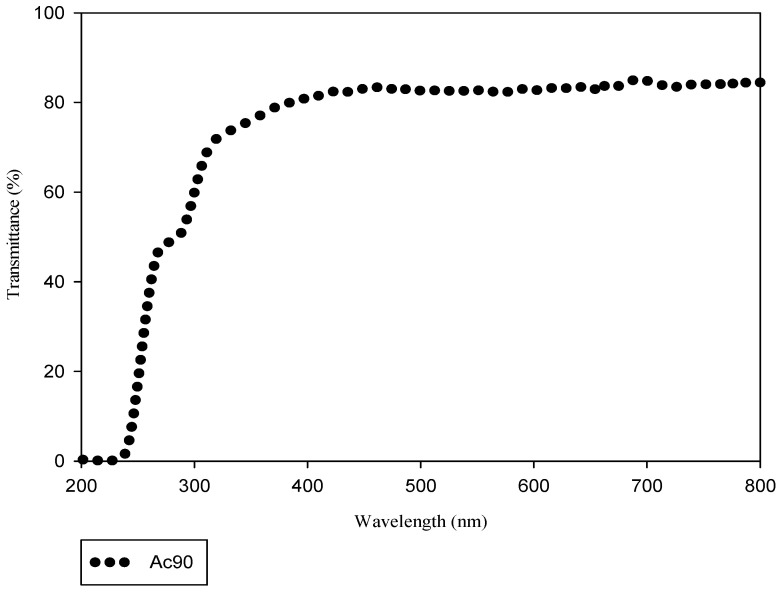
Light transmittance of Ac90 cellulose film.

The regenerated cellulose films exhibit good transparency, and their optical transmittance was investigated with a UV-vis spectrophotometer in the range of 200–800 nm (Figure 4) showing 80% of transmittance in the visible region. The result presented below is a mean of three measures of three different films. This result agreed with the literature that reports the good transparency of cellulose films [15,37].

### 3.6. IL Content in Films

In the washing step, cellulose gradually regenerates from dissolution through displacement of the IL by water. However, some IL is retained in the film structure as seen in the FTIR experiments. To quantify the amount of IL in films, an extraction method was used, in which film samples were placed in deuterium oxide (D_2_O) for 24 h, and the liquid analyzed by ^1^H-NMR using DMF as an internal standard. After extraction test, a FTIR spectra was acquired to prove that all IL was extracted to D_2_O. This spectrum is presented in Figure S1.2, in Supplementary Materials. The amount of IL in the extracts is calculated from a calibration curve constructed by measuring a determined mass of IL into D_2_O, using the same DMF internal standard. The calibration curve (Y = 1.1746 X) gives us the mass of IL as function of the integration value of the TMG peak in NMR spectra (Figure S2.3). TMG-acid solvent quantification is presented in Table 2 (see Supplementary Materials, Section S.2).

Quantification using ^1^H-NMR shows that a large quantity of IL was retained in the cellulose film structure. These values are comparable to others [11]. It was also found that cellulose dissolution temperature significantly influences the quantity of TMG based solvent retention, whereas the kind of acid (acetic versus propionic) does not play a critical role in this parameter. This increase of IL retention in films with the increase of dissolution temperature could be due to the substitution of some cellulose hydrogen bonds by cellulose solvent bonds, fixing the IL to cellulose structure. This difference in IL retention is quite evident when the mechanical properties of films were analyzed (see Section 3.9). A notorious plasticization effect due to the presence of IL is observed.

### 3.7. Thermal Properties

**Figure 5:**
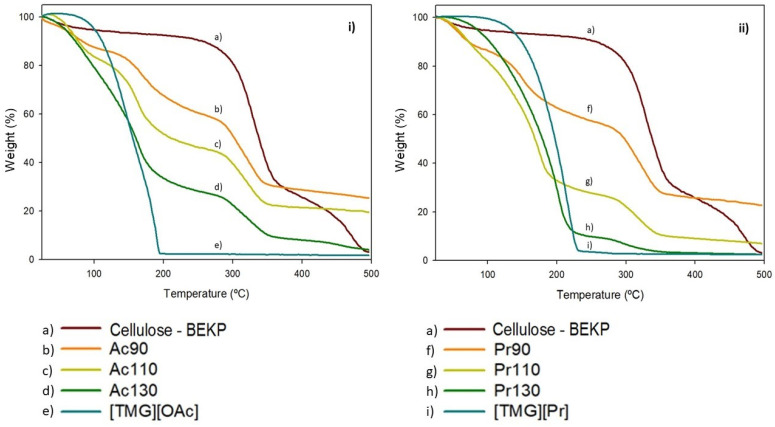
TGA curves of: (**i**) (a) cellulose pulp—BEKP, (b) Ac90, (c) Ac110, (d) Ac130 and (e) [TMG][OAc]; (**ii**) (a) cellulose pulp—BEKP, (f) Pr90, (g) Pr110 and (h) Pr130 and (i) [TMG][Pr].

Thermal properties of films obtained from different dissolution temperatures and with two TMG ionic liquids were determined by TGA and DSC curves. TGA curves of the regenerated cellulose films and cellulose pulp and TMG solvents for comparison are shown in Figure 5, as well as the corresponding values calculation in Table 3.

As shown in thermograms, TMG based solvents only have one weight loss stage and pristine cellulose has two weight loss stages. Cellulose films show three major distinct steps of weight loss but the first is probably due to water that remains inside the structure. Films with higher amounts of cellulose have a weight loss profile similar to cellulose and films with higher amounts of ionic liquid have profiles similar to the IL solvent. For films, the first stage corresponds to the water present in the film structure and the second step corresponds to the volatilization of the ionic liquid. The third stage is associated to the decomposition of cellulose. As seen in TGA curves, the onset temperature of IL solvent volatilization is lower than films for [TMG][OAc] and higher for [TMG][Pr]. This could be due to the solvent thermal stability because the [TMG][Pr] is more stable than [TMG][OAc]. On the other hand, the onset temperature of cellulose degradation is higher for BEKP cellulose pulp (373.3 °C) than for films (353.7–360.4 °C). During cellulose regeneration, its crystallinity dramatically changes, leading to a substantial decrease in its thermal stability.

As TGA could be used as a quantitative method and thermograms showed well defined steps, we used these results to estimate the amount of IL retained in films and the cellulose content, comparing with calculations by NMR (Table 2). For this propose, the temperature corresponding to 90% of mass of cellulose was targeted (257.3 °C). This temperature was considered as the beginning of cellulose degradation. Cellulose content was calculated by evaluation of mass loss between 257.3 °C and the residual mass at 500 °C. The calculation of IL content was the mass change between the beginning of IL degradation temperature 100 °C and 257.3 °C, which corresponds to a complete degradation of IL. If we only consider this step of IL degradation in the thermogram, the percentages of IL in films are very distinct from those obtained in extraction tests and NMR quantification. However, we observed that regenerated films present a high residual mass at 500 °C that is not observed in native cellulose or in the TMG ionic liquids. This fact, not very commented in works, has already been observed by Rogers that first describes the use of ionic liquid to dissolve cellose [9]. We assume that this residual mass could be due to ionic liquid that is associated with cellulose forming thermal stable structures. Considering this residual mass as IL, the results are presented in Table 4.

This TGA method for IL evaluation shows that IL content calculation by NMR and TGA presents similar values for all films, except for films with highest dissolution temperature (130 °C). These have a great amount of IL and low cellulose content. The discrepancy in IL content values with and without considering residual mass gives our theory some strength, believing that there is some IL chemically incorporated with cellulose chains, resisting to temperature degradation.

**Figure 6:**
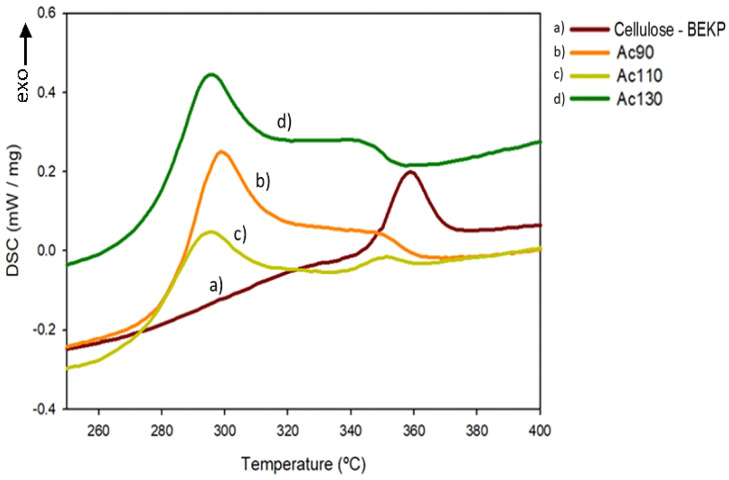
DSC thermograms of cellulose pulp and its films at the range of cellulose stability temperature of (**a**) cellulose—BEKP, (**b**) Ac90, (**c**) Ac110, (**d**) Ac130.

The DSC thermogram of cellulose/TMG-IL films show an endothermal transition in the range of 170–240 °C (Figure S3.2), depending on conjugated acid and dissolution temperature. A similar but less evident endothermal transition, around 120 °C, for cellulose/BMIMCl film solutions has already been reported by others [11]. For comparison, the cellulose thermogram (Figure S3.2) does not exhibit any thermal transition in this temperature range. These well-defined thermal events must be related to the TMG ionic liquid, particularly the evaporation process, as previous observed in the TGA. Regenerated cellulose films also show a small exothermic event (Figure 6) that, looking at the TGA, corresponds to a cellulose decomposition process. The KB model for cellulose decomposition [38,39] allows the occurrence of endothermic and exothermic events and this is observed by different authors [40,41]. In our case the regenerated celluloses follow an exothermic decomposition steps that correspond to the formation of char which are perceptible in the TGA of the regenerated samples [42] (Figure S3.3).

### 3.8. Crystallinity

**Figure 7:**
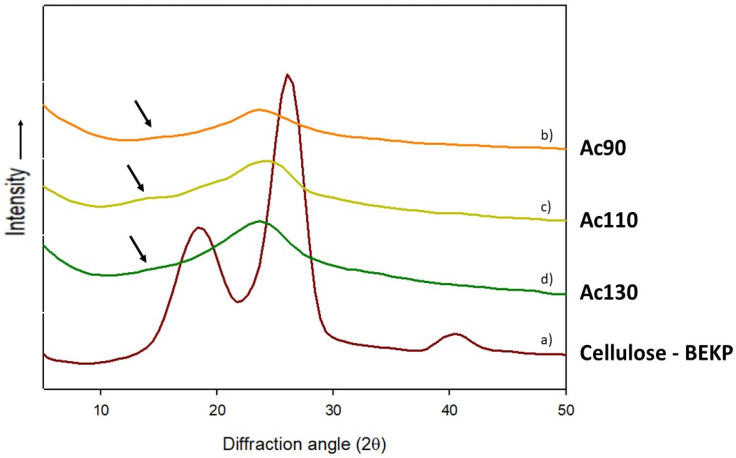
XRD profiles of (**a**) cellulose pulp—BEKP, (**b**) Ac90, (**c**) Ac110, (**d**) Ac130.

The XRD profiles of BEKP cellulose pulp and cellulose regenerated films are shown in Figure 7. Cellulose pulp presents diffraction peaks at around 18.6°, 26.2°, and a weak peak at 40.4°, being these diffraction angles higher than what is reported. Despite that, the XRD profile is in accordance with the literature, that shows the diffraction pattern corresponding to cellulose I [43]. The crystallinity index of raw cellulose pulp, calculated by Seagal’s method, is 53%, that is in accordance with literature values [29,43].

All regenerated cellulose films appear to have an amorphous structure. (Figure 6—spectra b, c, d) The XRD profiles showed a less intense and very broad peak at around 16° (black arrow in XRD profile) and a distinguishable peak at 24° which represents the same diffraction pattern of cellulose II crystalline structure [30]. It is clear from the results obtained during the dissolution and regeneration process that cellulose loose crystallinity and the small crystallinity developed during regeneration is typical of cellulose II. Similar works involving other ionic liquids have already reported the same behavior [35,44]. The values for the crystallinity index (Table 5) are, as expected, much lower than those of the native cellulose (Supplementary Materials, section S4), which is due to the break of intra and intermolecular hydrogen bonds of cellulose during the dissolution process, followed by a regeneration process that does not allow regaining of the original crystallinity [45]

The regenerated films produced with [TMG][OAc] have a lower crystallinity compared with the same films produced with [TMG][Pr]. For films produced with [TMG][OAc], the crystallinity increases with the increase of the dissolution temperature, whereas with [TMG][Pr] the crystallinity index is maintained. The increase of dissolution temperature led to a more efficient destruction of intermolecular interactions and thus to a better stacking of cellulose chains after regeneration. However, the increase of dissolution temperature and consequently break of hydrogen bonds, also promote a higher diffusion of ionic liquid solvent into cellulose chains, increasing the plasticization of cellulose.

### 3.9. Mechanical Properties

**Figure 8:**
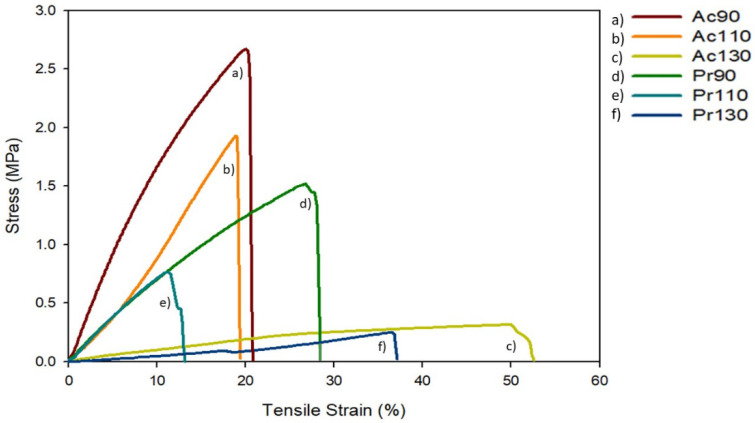
Typical tensile stress–strain curves of IL based cellulose films. (**a**) Ac90, (**b**) Ac110, (**c**) Ac130, (**d**) Pr90, (**e**) Pr110, (**f**) Pr130.

Mechanical properties were analyzed through tensile strength tests and the typical stress–strain curves of the films are shown in Figure 8. The results are also shown in Table 6.

Figure 8 and Table 6 present the effect of conjugated acid in ionic liquid and dissolution temperature on film properties. For two conjugated acids, the higher the dissolution temperature the lower the maximum stress and the higher the elongation at break. Comparing films of two acids at same dissolution temperature, the films with acetic acid are more resistant, presenting higher maximum stress (the double approx). This could be a result of the presence of more ionic liquid and higher plasticizing effect over the material, in case of propionic acid.

It is known that the tensile strength is dependent on the intermolecular interactions and crystallinity [30]. Due to the heterogeneity of cellulose chains, a lower degree of stacking and orientation in cellulose chains can lead to a poor distribution of stress, as well as a low elongation at break. With the increase of dissolution temperature, it was expected that these two properties would be enhanced. However, it was shown that with higher temperatures tensile strength was lower, only elongation at break increases. Even with the increase of crystallinity with higher dissolution temperature, the high amount of IL retention and consequently the lower quantity of cellulose in each film, which leads to a plasticization effect that overlaps the effect of crystallinity.

## 4. Conclusions

In this work, we developed a method to transform an industrial cellulose material to cellulose films by dissolution in a superbase (TMG) based system. The careful regeneration with water produces transparent and flexible films. The effect of temperature and ionic liquid on film properties was elucidated. The presence of TMG solvent in the cellulose film structure was confirmed by FTIR and extraction tests, revealing that the solvent varies from 45 to 95% wt. depending on acid and dissolution temperature used. SEM showed that cellulose films display a smooth and homogeneous surface and morphology. Thermal properties showed that cellulose stability in films was lower than in the raw cellulose and was confirmed by XRD, that suggested that most of the crystalline structure of cellulose is lost and the remaining changes from cellulose I to cellulose II after regeneration. With the increasing of temperature, crystallinity index also increases and improves elongation at break. These results validate a simple and effective method to prepare regenerated cellulose films with good properties, based on distillable and recyclable ionic liquid. Further work is in progress to perform a more environmentally friendly film by studying the recovery of the solvent and its use in a new dissolving process.

## Figures and Tables

**Table 1:** Cellulose solubility in tetramethylguanidine (TMG) based ionic liquids at 90 °C.

Sample	Solubility in TMG Based Ionic Liquids		
	[TMG][OAc]	[TMG][Pr]	[TMG][Fo]
MCC ^a^	5 wt.%	5 wt.%	Not soluble
BEKP ^b^	2 wt.%	2 wt.%	Not soluble

^a^ Avicel^®^ PH-101; ^b^ Industrial pulp.

**Table 2:** TMG based solvent quantification.

Film	IL (wt.%)
Ac90	45.1
Ac110	63.1
Ac130	88.6
Pr90	48.4
Pr110	61.7
Pr130	95.0

**Table 3:** Thermal properties of cellulose pulp, solvents and corresponding films.

Sample	DSC		TGA			DTG	
	T_s_ ^a^ (°C)	T_c_ ^b^ (°C)	T_onset_ ^c^ (°C)	T_end_ ^d^ (°C)	W_500_ ^e^ (%)	Solv_peak_ ^f^ (°C)	Cel_peak_ ^g^ (°C)
BEKP	-	358.9	-	355.1	3.0	-	373.3
[TMG][OAc]	205.9	-	140.8	193.9	1.7	189.2	-
[TMG][Pr]	213.6	-	159.1	222.7	2.3	215.4	-
Ac90	198.4	299.1	187.2	343.0	25.3	254.4	353.7
Ac110	212.6	295.6	157.3	339.3	19.5	251.9	357.3
Ac130	213.5	295.4	144.1	349.5	4.0	256.5	360.4
Pr90	203.4	338.4	146.5	343.5	22.6	256.4	357.7
Pr110	203.5	300.5	173.3	352.1	6.9	254.8	357.0
Pr130	224.4	302.9	204.6	354.0	2.4	255.0	355.2

^a^ solvent volatilization temperature; ^b^ cellulose stability temperature; ^c^ temperature of solvent volatilization; ^d^ onset temperature for cellulose stability; ^e^ residual mass at T = 500 °C; ^f^ peak of DTG corresponding to solvent volatilization step; ^g^ peak of DTG corresponding to cellulose degradation step.

**Table 4:** Cellulose and IL contents calculation by TGA.

Film	IL Content: Extraction Test (wt.%)	IL Content ^a^ (wt.%)	IL + Residual Mass ^b^ (wt.%)	Cellulose Content ^c^ (wt.%)
Ac90	45.1	27.5	49.8	34.6
Ac110	63.1	37.7	54.2	26.4
Ac130	88.6	58.9	65.5	12.9
Pr90	48.4	29.5	49.1	34.3
Pr110	61.7	54.7	61.6	20.3
Pr130	95.0	81.3	83.8	7.1

^a^ mass change between the beginning of IL degradation temperature 100 °C and 257.3 °C (beginning of cellulose degradation temperature); ^b^ the IL content step mass change was added to the residual mass at 500 °C; ^c^ the mass change between the beginning of cellulose degradation temperature (90% of mass, 257.3 °C) and the residual mass at 500 °C.

**Table 5:** Crystallinity index values.

Sample	Crystallinity Index (%)
Cellulose—BEKP	52.8
Ac90	9.2
Ac110	16.1
Ac130	27.1
Pr90	25.1
Pr110	21.1
Pr130	24.5

**Table 6:** Tensile-strength values calculated by a mean of five valid assays. Error associated to the presented values is the standard deviation (SD) of the five valid tests.

Film	Maximum Stress ± SD (MPa)	Elongation at Break ± SD (%)	Young’s Modulus ± SD (MPa)
Ac90	3.0 ± 0.5	28.7 ± 8.8	1.00 ± 0.10
Ac110	1.6 ± 0.3	18.9 ± 2.5	0.12 ± 0.02
Ac130	0.30 ± 0.03	46.9 ± 5.5	0.015 ± 0.002
Pr90	1.3 ± 0.4	19.5 ± 3.9	0.79 ± 0.09
Pr110	0.9 ± 0.2	14.7 ± 2.0	0.09 ± 0.01
Pr130	0.17 ± 0.05	33.9 ± 3.2	0.006 ± 0.002

## Data Availability

The authors confirm that the data supporting the findings of this study are available within the article and its Supplementary Materials.

## Supplementary Materials

The following are available online at https://www.mdpi.com/article/10.3390/polym13111767/s1. Sections: S1. [TMG][Pr] and film FTIRs, S2. Extraction tests, S3. Thermogravimetric analysis (TGA) and Differential Scanning Calorimetry (DSC) of solvents and films and S4. XRD index calculation and profiles.
