# Supplementary material for: Process Development for Flexible Films of Industrial Cellulose Pulp Using Superbase Ionic Liquids
Source: Polymers (Basel). 2021 May 28;13(11):1767. doi: 10.3390/polym13111767 (PMC8199285; doi:10.3390/polym13111767)
Supplement: Supplementary file 1 [file polymers-13-01767-s001.zip › polymers-1217982-supplementary.pdf]

polymers

MDPI

Article

Process Development for Flexible Films of Industrial Cellulose Pulp Using Superbase Ionic Liquids

Diana C. M. Ribeiro †, Rafael C. Rebelo †, Francesco De Bon, Jorge F. J. Coelho and Arménio C. Serra *

Centre for Mechanical Engineering, Materials and Processes (CEMMPRE), Department of Chemical Engineering, University of Coimbra, Rua Sílvio Lima-Polo II, 3030-790 Coimbra, Portugal; ribeiromdiana@gmail.com (D.C.M.R.); rafael.rebelo.94@gmail.com (R.C.R.); francesco.debon91@gmail.com (F.D.B.); jcoelho3@gmail.com (J.F.J.C.);

* Correspondence: aserra@eq.uc.pt

† These two authors contributed equally.

Citation: Ribeiro, D.C.M.; Rebelo, R.C.; De Bon, F.; Coelho, J.F.J.; Serra, A.C. Process Development for Flexible Films of Industrial Cellulose Pulp Using Superbase Ionic Liquids. *Polymers* **2021**, *13*, 1767. https://doi.org/10.3390/polym13111767

Academic Editor: Antonio Pizzi

Received: 26 April 2021
Accepted: 24 May 2021
Published: 28 May 2021

Publisher's Note: MDPI stays neutral with regard to jurisdictional claims in published maps and institutional affiliations.

Copyright: © 2021 by the authors. Licensee MDPI, Basel, Switzerland. This article is an open access article distributed under the terms and conditions of the Creative Commons Attribution (CC BY) license (http://creativecommons.org/licenses/by/4.0/).

Supplementary Information

Table of contents

S1. [TMG][Pr] and films FTIRs.

S2. Extraction tests.

S3. Thermogravimetric analysis (TGA) and Differential Scanning Calorimetry (DSC) of solvents and films.

S4. XRD index calculation and profiles.

S1. [TMG][Pr] and film FTIRs

1.1. FTIRs of IL and films

As seen previously for films with [TMG][OAc] solvent the characteristic peaks of cellulose like the stretching vibrations of hydroxyl group (-OH) at 1650 and 3300 cm^{-1} were observed too (Figure S1.1). Also, the [TMG][Pr] characteristic peaks are present in films after drying, showing the presence of it, after drying. There are no differences in spectrums in different solubilization's temperature of cellulose, proving that there are no chemical changes in cellulose structure, as for other conjugated acid.

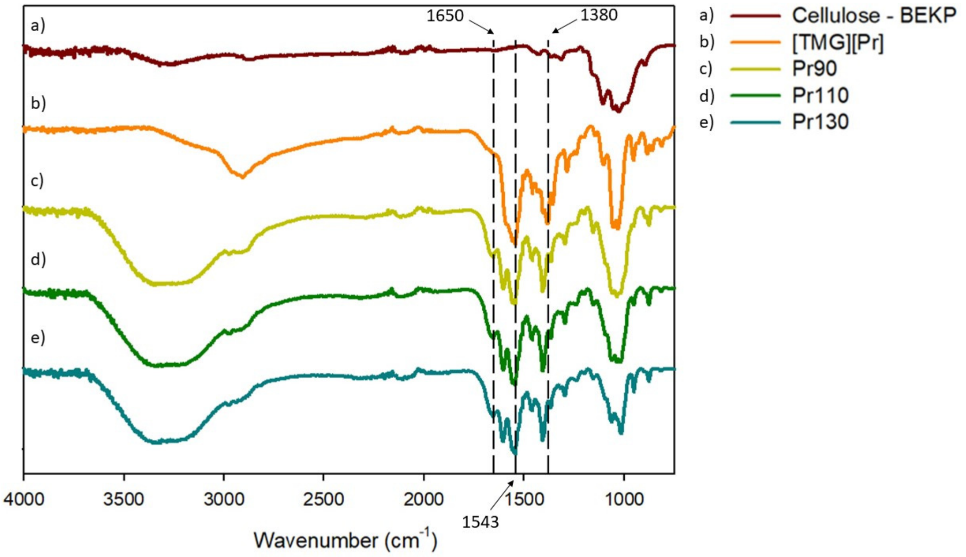

Figure 1. 1. FT-IR spectrum of (a) BEKP, (b) [TMG][Pr] and films (c) Pr90, (d) Pr110, (e) Pr130.

1.2. FTIR spectrum of a film after extraction test

In order to prove that all IL is extracted from film, in extraction test, a FTIR spectra was acquired after. The spectra of film before and after extraction are presented in Figure S1.2.

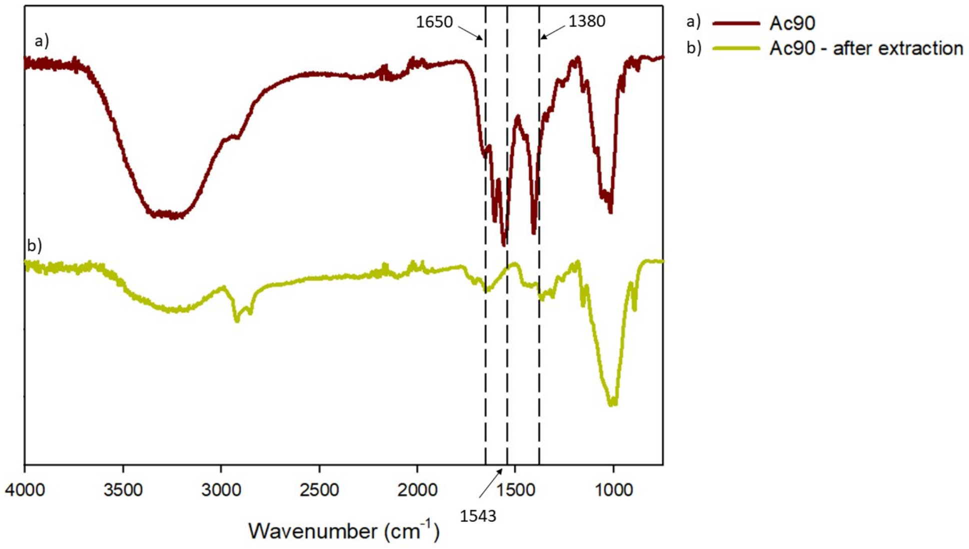

Figure 1. 2. FT-IR spectrum of (a) Ac90 and (b) Ac90 after extraction test.

As seen in Figure S1.2, the reference peaks of IL present in Ac90 (1380, 1543 and 1650 cm^{-1}) does not appear in Ac90 – after extraction spectra, that indicates the whole IL presented in film was extracted to D_2O, and extraction was full.

S2. Extraction tests

2.1. ^1H NMR spectra of the TMG based solvents extracted from films

^1H NMR was performed for all extraction tests from different cellulose films. The follow figures (Figure S2.1 and S2.2) represent an example of the signals that were choose to calculations.

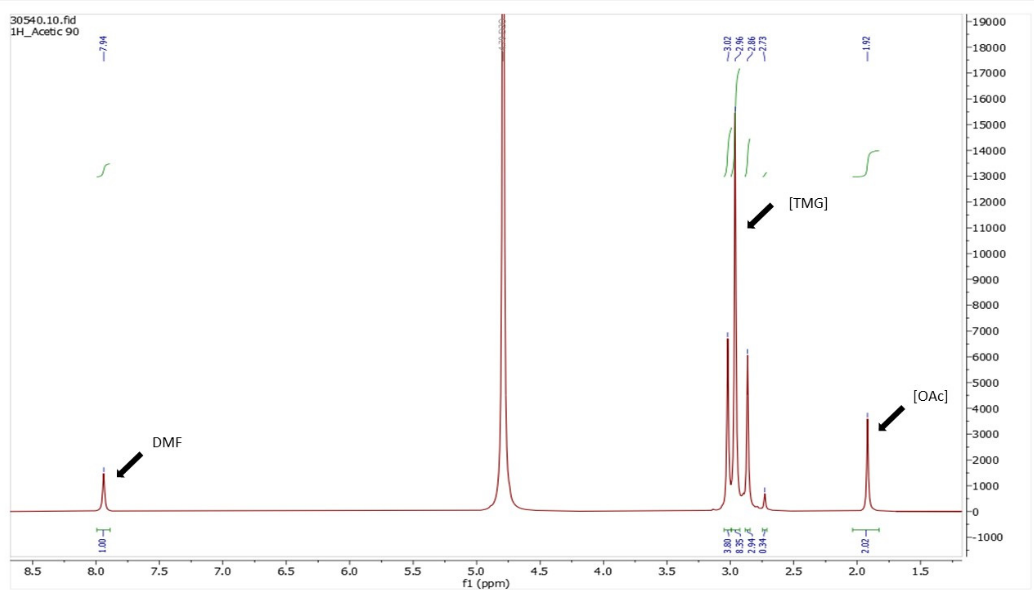

Figure 2. 1. ^{1}H-NMR spectra of IL extracted from the cellulose film (Ac90) in D_2O.

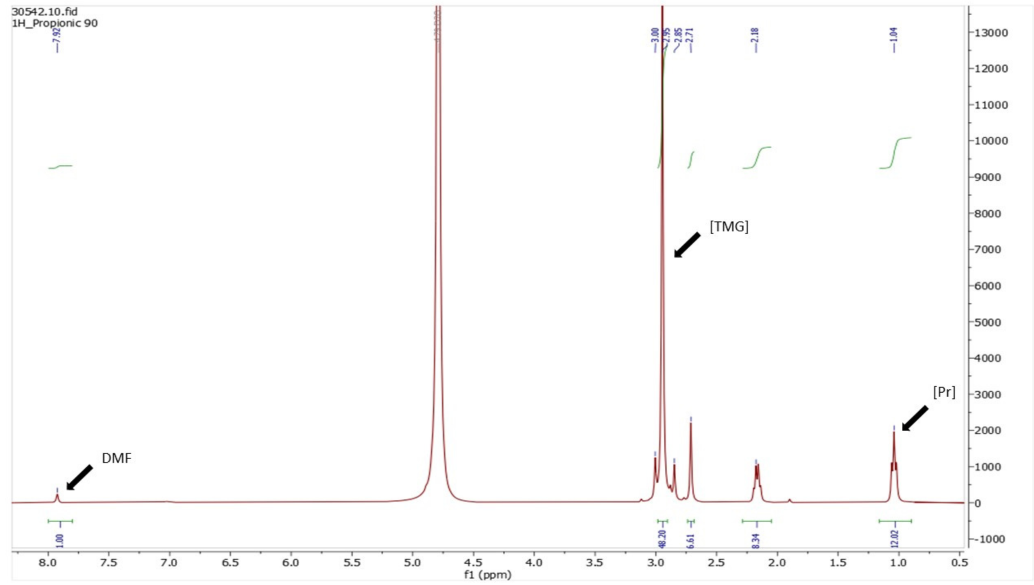

Figure 2. 2. ^{1}H-NMR spectra of IL extracted from the cellulose film (Pr90) in D_2O.

2.2. Calibration curve

Once some peaks of interest (TMG at 2.96ppm) are not isolated and very well defined in NMR spectrum and its integration is not so easy to calculate and it could have a significant error, we prepare a calibration curve. To achieve the calibration curve three measured contents of IL and DMF intern standard were prepared in D2O, and the relation between the DMF signal integration corresponding to the -CH, at 7.92 ppm and the integration of the TMG protons at 2.95 ppm. This correlation (Figure S2.3) is a directly proportional straight with an equation Y = 1.1746 X, where Y is the amount of IL present in films and X is the integration of TMG peak integration.

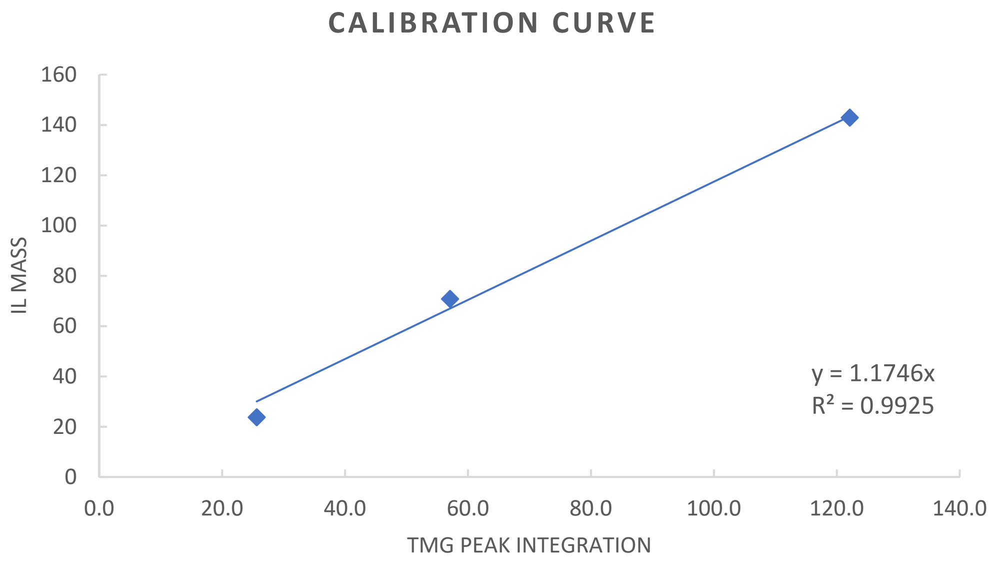

Figure 2. 3. Calibration curve calculated by the correlation between the TMG peak integration, in relation of DMF peak in NMR spectrum, and a measured IL mass.

Besides the coefficient of determination, R2, is very high and so the correlation and fit between the points measured are very good, the Table S2.1 presents an example of one point of calibration curve, where can be proved that this method is a very well-defined approximation of the quantity of IL present.

Table 2. 1 Calibration curve fitting.

IL-1	NMR peak [a]	H [b]	n (mol)	Mass Calculation [c] (mg)	Mass Measured [d] (mg)
DMF	1.0000	1	0.063	-	4.61
Acetic acid	6.1200	3	0.129	7.73	-
TMG	25.6200	12	0.135	15.51	-
IL	-	-	-	Sum: 23.2	23.8

[a] integration value of peak in NMR spectrum; [b] number of protons that correspond the peak; [c] mass of each IL compound calculated by the calculation of molar quantity (mol), thus relationship between the NMR peaks integrations; [d] mass measured of each compound in NMR solution

As seen in Table S2.1, the correlation between the mass calculation of IL (23.2 mg) and the real mass measured (23.8 mg) gives us a good approximation of the IL quantity and so the extracted IL from cellulose films.

2.3. IL content calculation

The IL content calculation was obtained by the calibration curve calculated previously. Thereby, the integration of TMG solvent peak was used to calculate the mass of IL extracted from films. An example of this process is presented on Table S2.2, for Ac90, that NMR spectrum is already presented on Figure S2.1. Note that the mass of IL extracted in test was not the total mass of IL in NMR tube, once the test is performed in 2ml of D2O and only 700μl are used in NMR test. For that reason, is necessary to do a correction of mass extracted for 2 ml solution.

Table 2. 2 IL content in cellulose film, Ac90.

Ac90	NMR Peak [a]	H [b]	IL' Mass Calculation [c] (mg)	IL' Mass Extracted [d] (mg)	Film Initial Mass [e] (mg)	% IL [f]
DMF	1.0000	1	-	-	-	-
TMG	8.3500	12	-	-	-	-
IL-extract	-	-	9.8	28.0	62.02	45.1

[a] integration value of peak in NMR spectrum; [b] number of protons that correspond the peak; [c] mass of IL extracted calculated by the calibration curve, in NMR tube; [d] mass of IL extracted for 2ml of solvent; [e] films' mass before extraction test; [f] percentage of IL extracted from film.

Scheme 3. Thermogravimetric analysis (TGA) and Differential Scanning Calorimetry (DSC) of solvents and films. .

Thermograms of solvents shown that TMG solvents are thermally stable at least at 100 °C. Also, the conjugated acid modifies the thermal stability too. That was expected that with the increase of acid chain, the stability increases. The decreasing physical and mechanical properties of films with higher dissolution temperature could be derived of some solvent degradation too, especially in acetic case.

As seen in Figure S3.2, cellulose thermogram does not exhibit any thermal transition in solvent volatilization temperature range, whereas cellulose composite films presented an endothermal transition corresponding to the solvent volatilization in films.

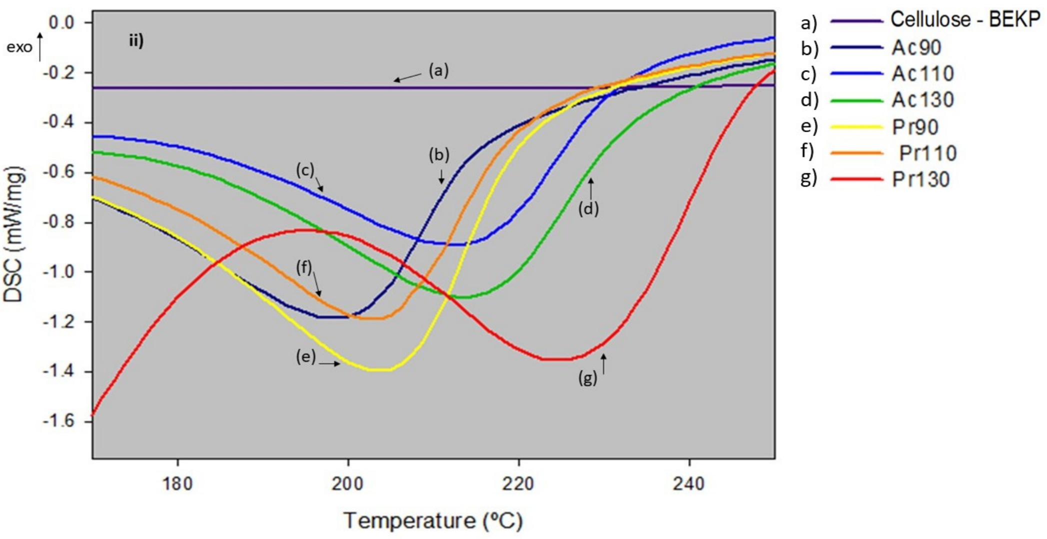

Figure 3. 2. DSC thermograms of cellulose pulp and its films at solvent volatilization temperature. (a) Cellulose - BEKP, (b) Ac90, (c) Ac110, (d) Ac130, (e) Pr90, (f) Pr110 and (g) Pr130.

In cellulose decomposition temperature, also in propionic based films, the thermal stability of cellulose decreases in reference of cellulose pulp. These results are in accordance with the results obtained for Acetic based films and with the previously reported in literature too. As shown in Figure S3.3 the peak of thermal transition corresponding of cellulose decomposition is approximately 50-60 degrees lower than the cellulose pulp.

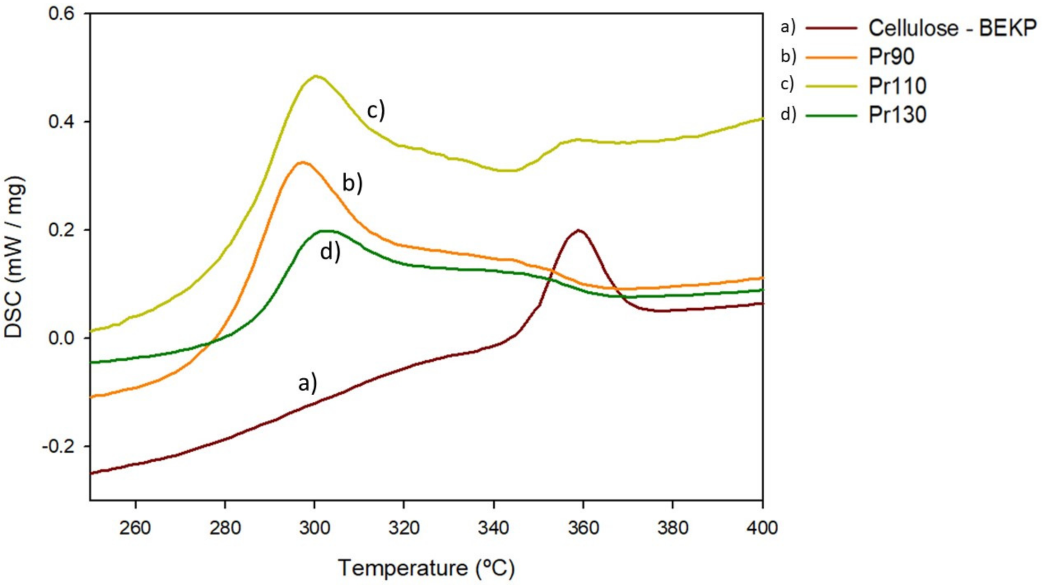

Figure 3. 3. DSC thermograms of cellulose pulp and its films at cellulose decomposition temperature. (a) Cellulose - BEKP, (b) Pr90, (c) Pr110, (d) Pr130.

S4. XRD profiles

To trace the XRD profiles, the raw data was processing using a smooth 2D data program from SigmaPlot® (version 10.0) by a negative exponential smoother of 4th degree and a sampling proportion of 0.100. After that, the profiles were traced and the crystallinity index calculated.

As seen in Figure S4.1, an example for the XRD profiles of cellulose composite films, these has not the two peaks of cellulose absolutely defined, so to use the Segal's method the smaller peak, corresponding the broad peak in cellulose profile, evidenced by the arrow in XRD profile, is the minimum peak intensity (I_{am}). The main peak is completely well defined and was not necessary an approximation for the value of I_{002}.

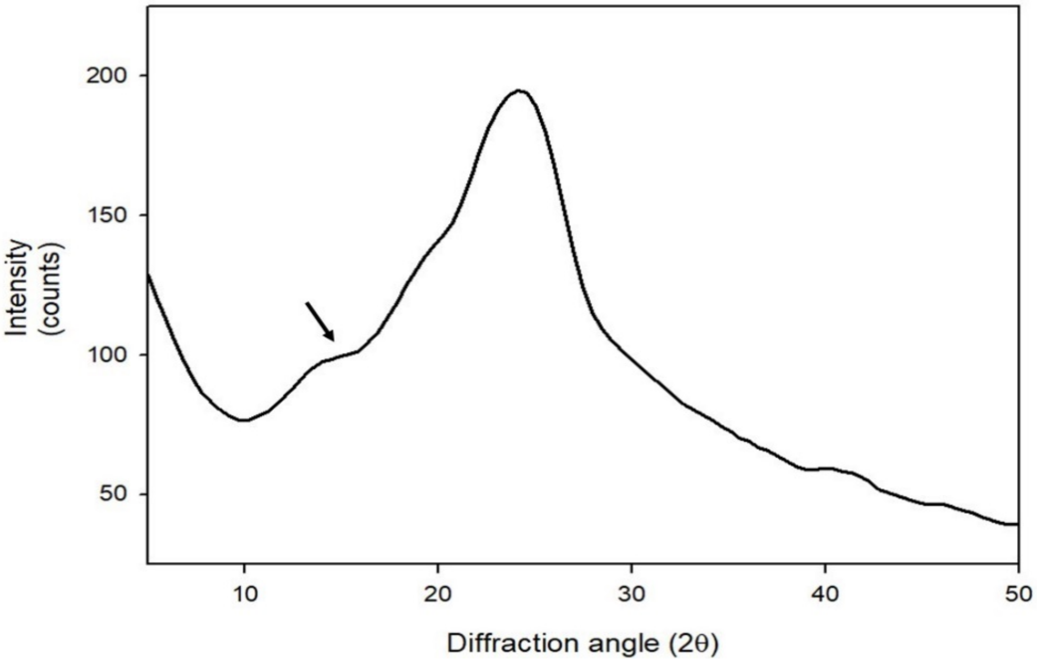

Figure 4. 1. XRD profile of Ac110, for calculation of crystallinity index. The black arrow corresponds to the I_{am} and the I_{002} is the intensity of the main peak.

The XRD profiles of cellulose composite films, from [TMG][Pr] solvent, are presented in Figure S4.2 too.

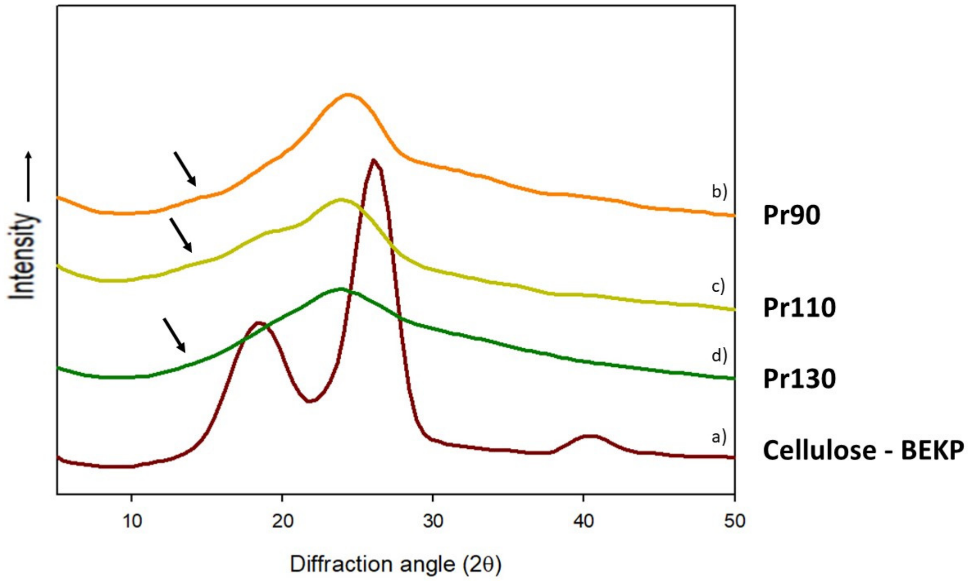

Figure 4. 2. XRD profiles of (a) Cellulose pulp - BEKP, (b) Pr90, (c) Pr110, (d) Pr130.

As composite films from [TMG][OAc] solvent, also the films from propionic acid as conjugated of TMG shown the same XRD profile pattern corresponding to the crystallinity pattern of cellulose II. The dissolution and regeneration bath with this solvent transform cellulose from cellulose I to cellulose II too. The films' crystallinity index was also lower than the cellulose pulp, as expected.
